# Characterization of Emissions from Carbon Dioxide Laser Cutting Acrylic Plastics

**DOI:** 10.1021/acs.chas.3c00013

**Published:** 2023-06-22

**Authors:** Alejandro Munoz, Jacob Schmidt, I. H. Mel Suffet, Candace Su-Jung Tsai

**Affiliations:** † Department of Environmental Health Sciences, Fielding School of Public Health, University of California—Los Angeles, Los Angeles, California 90095-1735, United States; ‡ Samueli School of Engineering, University of California—Los Angeles, Los Angeles, California 90095-1735, United States

**Keywords:** laser cutter emissions, methyl methacrylate, poly(methyl methacrylate), particulate matter, nanoparticles, microplastics, nanoplastics

## Abstract

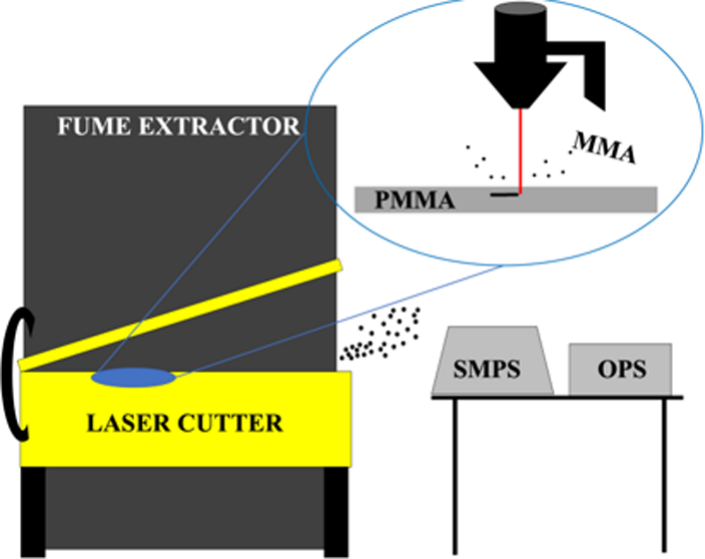

Carbon dioxide laser cutters are used to cut and engrave on various types of materials, including metals, wood, and plastics. Although many are equipped with fume extractors for removing airborne substances generated during laser cutting, gases and particulate matter can be released upon opening the lid after completion. This study focused on investigating laser cutting acrylic sheets and associated emissions. Real-time instruments were utilized to monitor both particulate concentrations and size distributions, while the patented Tsai diffusion sampler was used to collect particulate samples on a polycarbonate membrane and transmission electron microscopy (TEM) grid. Identification of released gases consisted of the use of gas sampling with Teflon gas bags followed by analysis using gas chromatography-mass spectrometry (GC-MS). A portable ambient infrared air analyzer was used to quantify the concentrations of the chemicals released by laser cutting activities. The results of the study found that a significant concentration of particulate matter, including nanoplastic particles ranging 15.4–86 nm in particle sizes, and microplastics with agglomerates were released each time the laser cutter lid was opened and were observed to gradually increase in concentration for a period of at least 20 min after the completion of a cut. The GC-MS gaseous samples primarily contained methyl methacrylate at a low level close to the detection limit of the infrared air analyzer.

## Introduction

One of the fabrication processes that is growing in popularity is the use of carbon dioxide (CO_2_) laser cutters.^1^ Laser cutters are used in various industries because of their ability to cut and engrave different materials at a high degree of accuracy and precision without having to constantly change tools due to wear from repetitive use.^2,3^ Because of their compact size, low cost, and reliability, these laser cutters can be placed in small workspaces, classrooms, offices, and other convenient locations. Compact laser cutters produce high-intensity infrared light beams that can reach hundreds of watts/cm^2^ of power density to achieve cutting and engraving material such as glass, metals, polymers, and wood.^1−4^ The high-intensity beams cause melting, evaporation, and volatilization of the material, which in turn generate emissions in the form of gases, chemical vapors, and particulate matter that are referred to as laser-generated air contaminants (LGACs).^1,2,4,5^ The types of LGACs released from laser cutting will depend on various factors such as the type of material used, the speed and power at which the laser cutter is operated, and the duration of the cut.^5−7^

Local exhaust filtration systems, such as fume extractors, are available to use with laser cutters that are designed to reduce the LGACs that are emitted from cutting different materials.^8^ The fume extractors are generally equipped with a series of filters, which are primarily for the filtration of particulates, and adsorbent materials, such as activated carbon, which are effective in reducing chemical emissions.^8^ However, since some of the systems are connected through the back portion of the laser cutters, there is still potential for emissions to leak out through the front when the lid is opened after a cut has been completed. The particle and chemical emissions that escape through the laser cutter lid once the fume extractor systems have been turned off have yet to be quantified and characterized for laser cutting several types of materials, including poly(methyl methacrylate) (PMMA).

Among the most used type of plastic material within carbon dioxide laser cutters is poly(methyl methacrylate) (PMMA), otherwise known as acrylic.^9^ PMMA is a thermoplastic polymer that is popular due to its low cost as well as chemical and physical properties that result in high-quality cuts.^9−12^ It is used in dentistry, the production of electronics, greenhouses, and various other products.^9^ PMMA has a rigid structure that responds well to the energy produced by CO_2_ laser beams, and as a result, it can absorb laser energy quickly, resulting in a faster and more precise cut.^9−12^ When exposed to the high heat of the laser, PMMA undergoes thermal degradation and can release methyl methacrylate (MMA), ethyl acrylate, phenols, and polycyclic aromatic hydrocarbons (PAHs) in addition to particulate matter.^1,6,13^ PAHs are a group of organic compounds that can be found in the air as a result of the incomplete combustion of organic materials.^14−17^ Although the exposure and associated health effects to PAHs from CO_2_ laser cutting have not been evaluated, certain types of PAHs have been identified as carcinogenic, mutagenic, and teratogenic to humans.^14−17^ Particulate matter refers to the solid and liquid particles suspended in the air that can be hazardous based on their size and composition.^18^ Particulates with a physical diameter of less than 100 nm are referred to as nanoparticles (NPs) and can be toxic in high concentrations due to the larger surface area to volume ratio compared to larger particles of the same mass.^5,19,20^ NPs that enter the body through the inhalation pathway can deposit deep into the lungs and can cause inflammation and oxidative stress in the lungs and lead to respiratory diseases and other health problems.^14,19−21^ The toxicity of the NPs has been shown to vary based on the size, shape, and chemical composition, with smaller particles having a higher degree of toxicity.^15,19^ Exposures and health effects related to NPs produced from CO_2_ laser cutting have yet to be evaluated. Microplastics and nanoplastics are other forms of particles in micro sizes and nanoscale, which would be emitted from cutting acrylic plastics. Microplastics are particulates that are formed from the degradation or processing of plastic materials that are less than 5 mm in diameter.^22^ Nanoplastics are those further degraded or fragmented from microplastics with size ranging from 1 nm to 1 mm.^23^

A study by Haferkamp et al. found that laser cutting PMMA generated a higher distribution of smaller-sized particles when compared to the other types of plastics, as well as high levels of PAHs.^5^ The peak diameter for PMMA-generated aerosols occurred at about 0.05 μm (50 nm).^5^ However, the rate of particular emissions was found to be relatively low when compared to other types of plastics.^5^ The above study does not make reference to the total concentration of particulates and chemical emissions at various parts of the laser cutting process, which would be important to note in order to develop a more efficient control measure. Another study by Kiefer and Moss found that laser cutting PMMA generated particles within a 25 W CO_2_ laser cutter enclosure that was more than 10 times the measured background levels.^24^ This study focused on the LGACs produced within the enclosure of the laser cutter but does not refer to the total concentration that escapes from the lid during various phases of the laser cutting process. In addition, both studies focused on relatively short cutting times and did not characterize the emitted substances. In another study, the use of a laser cutter resulted in emissions of VOCs and ultrafine particles during 400 s following the cutting operations.^25^ Since emissions are dependent on an abundance of factors such as the process performed, laser intensity, laser power, laser speed, and duration of the task, particulate emissions generated at different operating processes will be of concern, especially if longer cuts generate a higher concentration of LGACs.^6^ This study is the first to investigate the total and size-fractioned concentrations of particles and chemicals emitted from laser cutting at various phases of the laser cutting process for longer cut times, as well as characterizing the emitted particles including micro and nanoplastic particles using the sampler designed specifically for collecting nanoscale particles.

## Methods

### Study Design

This project was conducted to investigate the particulate and gas emissions released from cutting PMMA sheets with the use of a 60 W compact laser cutter. Throughout the study, a total of six laser cutting procedures were observed and monitored. The first part of the study focused on the particulate matter emitted during and after the laser cutting process. Particulate size and range distributions were monitored with the use of real-time monitoring instruments throughout the laser cutting operation. Samples of particulate matter were collected using the Tsai diffusion sampler (TDS) and analyzed with microscopy.^26^ The second part of the study focused on the chemical gas emissions released during the lid opening upon the completion of the cutting. Gas samples were collected using 1 L Teflon gas bags and were analyzed using gas chromatography-mass spectrometry (GC-MS) for chemical identification. After the chemical identification, a portable infrared (IR) analyzer, which uses an infrared spectrophotometer, was used to measure the concentration in real time. The portable analyzer includes a library of gases and can be preset to detect certain chemicals at different wavelengths. The conditions for each experiment were kept as similar as possible, changing only the waiting time before turning off the fume extractor and opening the lid of the laser after each cut. The time durations were chosen based on observations of the operation of the laser cutter by the users.

### Facility and Equipment

The workplace that hosts the laser cutter in this study has two available CO_2_ laser cutters. A desk is situated in between both laser cutters, which is where individuals that use the laser cutters can create designs and monitor their projects. In addition to the studied laser cutter, there are about 21 small-scale 3D printers located at the opposite side of the wall available for use by visitors to the facility, as well as a second laser cutter that is ∼4 feet (1.2 m) away from the primary laser cutter used during the experimentation. The 3D printers located in the facility primarily use the materials acrylonitrile butadiene styrene (ABS) and poly(lactic acid) (PLA) and are located at a distance of ∼40 feet (12 m) from the laser cutter area. The area of the space is ∼5250 square feet (487 m^2^) and has an average ceiling height of 8 feet (2.4 m). The facility is equipped with two air handling units, which combine for a total of 6.5 air exchanges per hour when they are used simultaneously. The entrance to the facility is located near an industrialized area that includes a warehouse and machine shop. The air duct used for the main ventilation system is located near the front entrance of the facility, facing the industrialized alleyway. This leads to potential concern of outside pollutants, such as diesel particulates, being ventilated indoors through both the ventilation system and the doors each time they are opened.

### Laser Cutter and Materials

A 60 W laser cutter (Universal Laser Systems, VersaLaser—VLS6.60), which produces a 10.6 μ infrared laser, was used to cut a set design into PMMA sheets measured to be 0.125 inches (0.32 cm) thick. The intensity of the laser was set at the maximum power to ensure a complete cut of the sheets. The manufacturer user interface software was used to ensure that both the design and the cutting time were kept constant. A BOFA Fume Extractor (AD 1000 IQ) is connected to the rear portion of the laser cutter as a local exhaust ventilation system to reduce the pollutants emitted from the process. The fume extractor is designed with a borosilicate prefilter that captures large particulates (95%, 0.9 μm), a combined filter that captures smaller particulates with a HEPA Filter (99.997%, 0.3 μm), and an activated carbon filter to filter out chemical gas emissions. As per facility requirements, the fume extractor was turned on prior to starting any cut and was kept on for varying amounts of time after the completion of a cut.

### Experimental Process

#### Particulate Monitoring Equipment and Data Analysis

The total concentration and size range distribution of particulate emissions were monitored throughout the entire laser cutting process. The instruments were set up on a cart directly in front of the laser cutter as shown in Figure S1A,B. A nanoscan scanning mobility particle sizer (Nanoscan SMPS, TSI Model 3910, Shoreview, Minnesota, 10–420 nm, 13 channels, concentrations 0–10^6^ particles/cm^3^), abbreviated as SMPS in this study, was used to continuously monitor particles ranging from 10 to 420 nm at 1 min intervals. Tygon tubing was connected to the cyclone inlet of the instrument and placed 2.5 inches (6.23 cm) from the lid of the laser cutter as shown in Figure S1B. An optical particle sizer (OPS, TSI Model 3330, Shoreview, Minnesota, 0.3–10 μm, 16 channels) was used to continuously monitor particles ranging from 0.3 to 10 μm in 1 min intervals. Similarly, Tygon tubing was also connected to the inlet nozzle and placed 2.5 inches (6.4 cm) from the lid of the laser cutter. An additional OPS was placed on a nearby desk, which was measured to be 7 feet (2.1 m) away from the studied laser cutter and monitored particulate matter during the entire operation. Lastly, outside concentrations were monitored with an OPS for a duration of 2 h during the same period in which experiments were conducted. The data that was collected from the SMPS and OPS was downloaded into the aerosol instrument manager (AIM) software and converted into a CSV file. The graphs were created using Microsoft Excel. Data analysis included the two-sample *t*-test, Pearson’s test for correlation, and the analysis of variance (ANOVA) test. A one-way analysis of variance (ANOVA) test was used to analyze the difference in the group mean total particulate concentration of the data collected during the background, laser cutting, lid opening, and post-background. The null hypothesis assumed that the mean total concentration would not differ among each portion of the laser cutting activity (H_0_: μ_1_ = μ_2_ = μ_3_ = μ_4_). Differences were found to be significant at a *p*-value < 0.05. A Pearson’s test for correlation was used to test the correlation between the particulate size range distributions during each experimental period. A Pearson correlation coefficient of 1.00 signifies a strong positive correlation between the particle distribution and portion of the laser cutting activity. The two-sample *t*-test was used to test only between the background and post-background phases to determine if there was a significant change in particulate concentration that occurred throughout the experimental period. The change was significant at a *p*-value less than 0.05.

#### Monitoring Procedure and Sampling Methods

Particle emissions were sampled and collected throughout the entire laser cutting procedure. The monitoring was divided into four portions: background (20 min), cutting (10 min), lid opening, and post-background (20 min). The only factor that was changed was the amount of time that was waited before the fume extractor was turned off and the lid was opened (Method 1—0 s, Method 2—30 s, Method 3—1 min). The amount of time waited was based on the observation of the amount of time users would wait before opening the lid of the laser cutter as listed in Table S1.

#### Particle Sampling and Analytical Methods

TDS was used to collect samples of particulate matter in the respirable size range. The TDS can be used to collect the particles directly onto a polycarbonate (PC) membrane filter and transmission electron microscopy (TEM) grid.^26^ The membrane filter used was a 25 mm diameter, 0.22 μm pore size PC membrane, and a TEM-copper grid in 400 mesh with the carbon-coated film was placed at the center of the filter.^26^ The TDS was used with a Gilian GilAir Plus sampling pump calibrated to a flow rate of 0.9 L/min. The TDS was placed facing horizontally at 2.5 inches (6.4 cm) from the laser cutter lid. The particles collected on the TEM grid were analyzed using an FEI Tecnai T12 transmission electron microscope (Tecnai T12, FEI, Oregon) operated at an electron tension of 120 kV at various levels of magnification.

#### Gas Sampling and Analysis

Samples of gas emissions were collected during the lid opening using a manual 1 L pump along with 1 L Teflon gas bags from Jensen Inert Products (Coral Springs, FL). The chemical gas samples were extracted from the bag using solid phase microextraction (SPME) that is based on the principle of adsorption and absorption and is widely used in the analysis of environmental pollutants in water, soil, and air.^27,28^ In the SPME method, the analytes are extracted from the gaseous media by using a coated fiber within a syringe.^27,28^ The analyte is then injected into a gas chromatograph (Varian 450, Varian Inc., California) equipped with a mass spectrometer (Varian 220, Varian Inc., California). During the experimental process, first, a 1 cm 50/30 μm DVB/Carboxen/PDMS sold phase method extraction (SPME) fiber from Supelco (Bellefonte, Pennsylvania) was used to extract the sample within 15 min at room temperature from a 1 L Teflon Air Bag. The fiber is a bipolar 50/30 fiber, and was chosen because it can extract a large variety of chemicals for a broad spectrum analysis. The GC Column used was 60 m, 0.25 mm diameter, DB-5MS (0.25 μm) stationary phase from Agilent Technologies, Inc. (Santa Clara, California). The SPME fiber was introduced into the gas chromatographic injector at 270 °C for 15 min. The GC column oven temperature was programmed with 50 °C for 2 min and then an 8 °C per min rate to 270 °C and held for 6.5 min. The GC column flow rate was 1.0 mL/min. The system can then identify chemical constituents contained in the sample by comparison of the acquired mass spectrum from a NIST library of Mass Spectrum.

To assess the real-time concentration of any identified chemical gases, a portable infrared ambient air analyzer (MIRAN 205B Series SapphIRe, ThermoElectron, Massachusetts) was utilized. The SapphIRe was factory-calibrated and configured to monitor the chemical methyl methacrylate (MMA) at two different wavelengths (10.7 and 12.3 μm). The detection limit for MMA is 0.4 parts per million (ppm). The SapphIRe measures the concentration (ppm) of the selected chemical gas every 30 s in real time.

## Results and Discussion

### Airborne Particles Released from Laser Cutting Activities

**Figure 1:**
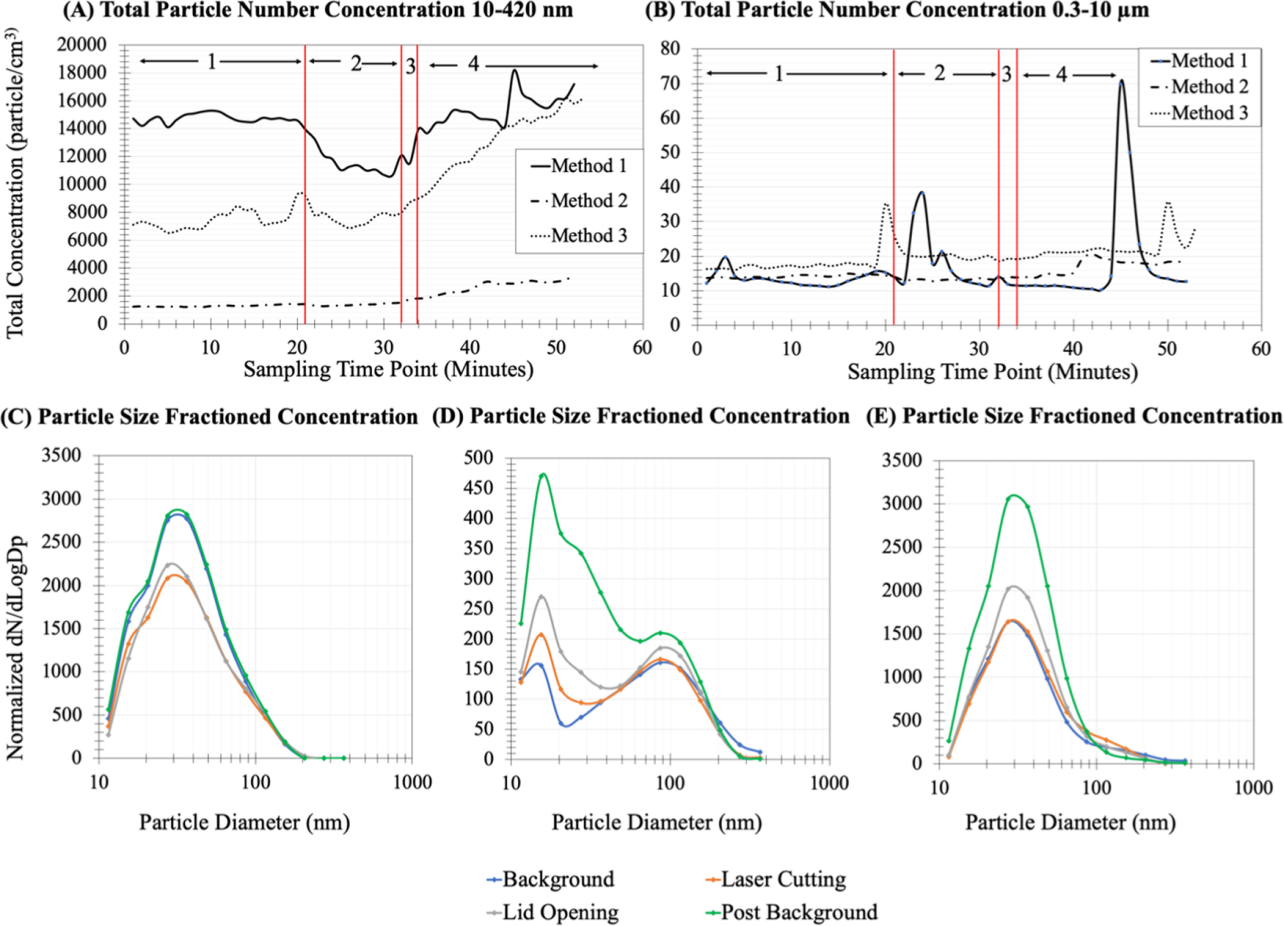
Total concentration of particulate matter during four different periods of experimentation: (1) background, (2) laser cutting, (3) lid opening, and (4) post-background. (A) Total concentration of particulates with diameters ranging from 10 to 420 nm as measured by the Nanoscan SMPS. (B) Total concentration of particulates with diameters ranging from 0.3 to 10 μm as measured by the OPS. (C–E) Particle size fractioned concentrations (d*N*/d LogDp) measured by the NanoScan SMPS during each phase of the laser cutting activity. (C) Size distribution for Method 1, (D) size distribution for Method 2, and (E) size distribution for Method 3. Note: Concentrations are normalized data.

Changes in the total particle concentration and distribution were observed during the four stages of the laser cutting activities (background, laser cutting, lid opening, and post-background). Figure 1A,B illustrates the changes in total particulate concentration for each experimental method as measured by real-time instruments. Although there was an average of five small 3D printers in operation on the far side of the workplace during each of the experimental periods, we did not observe measurable emission migrated from 3D printers to the laser cutter. However, based on the data that was collected, it was observed that the second laser cutter, 4 feet (1.2 m) distance, was more likely to influence spikes in particulate concentration. The use of the second laser cutter, which is ∼4 feet (1.2 m) from the studied laser cutter and 6 feet (1.8 m) from the OPS, occasionally corresponded with several spikes in concentrations, such as the ones seen during the background and laser cutting phases shown in Figure 1B. However, the time period during cutting and lid opening of the primary laser cutter Method 1 contributed clearly to the peak in Figure 1B.

### Total Concentration and Distribution in 10–420-nm-Sized Particles

Based on the graph illustrated in Figure 1A, a steady, gradual increase in total particulate concentrations was observed during the 20 min post-background period after the laser cutting had been completed, and the fume exhaust was turned off. The concentration of each method was an average of two laser cutting trials, and all six individual trial data were presented in Figure S2 with experimental information shown in Table S2. An ANOVA statistical test was used to determine that there was a significant difference between the total concentration means of each phase of laser cutting activities (Method 1: *p*-value = 1.75 × 10^–11^; Method 2: *p*-value = 4.4 × 10^–17^; Method 3: *p*-value = 3.9 × 10^–16^). A follow-up *t*-test between background and post-background total particle concentrations determined that there were only significant differences between the total background and post-background concentrations in Methods 2 and 3 (*p*-value = 2.3 × 10^–14^ and 1.8 × 10^–13^, respectively). A significant increase in concentrations for particulates in the 10–420 nm size range was observed to occur after the opening of the lid; during the post-background period, small particulates were being released each time the lid was opened after a cut was complete. It was also observed that the highest concentration of particles was measured during the post-background period across all three experimental methods (Method 1—18 143 particles/cm^3^; Method 2—3515 particles/cm^3^; Method 3—16 190 particles/cm^3^), thus further supporting the hypothesis that particulate matters were escaping after the opening of the lid even with the use of a fume extractor during cutting.

This gradual increase of concentration during the post-background period was accumulation of emitted particles from the cutting. The particle distribution graphs (Figure 1C–E) demonstrated that the particle size distribution remained the same throughout the entire experimental period. Statistical analysis (Pearson’s correlation) used to compare the particle size distributions during each of the portions of the laser cutting confirmed a high degree of correlation between each of the phases of the laser cutting activity of Methods 1, 2, and 3 in all particulate sizes measured by the NanoScan SMPS and OPS (Tables S3–S10 presenting statistical analysis data). Thus, the correlation between the distribution of particle sizes can be taken to mean that the laser lid opening contributed to accumulation of particles and did not result in the emission of particulate matter different in sizes from that which was already observed in the background measurements. There was a noticeable increase in particulate emissions during the post-background period among all three of the experimental methods. Particles ranging from 27.4 to 36.4 nm were found to have the highest concentration during all stages of laser cutting activities. The highest peak concentrations for particles in this range were 2821 and 3057 particles/cm^3^ for Methods 1 and 3, respectively, during the post-background phase of the laser cutting activity. However, during experimental Method 2, there was a bimodal distribution observed. The distribution was highest for particles with diameters of 15.4 and 86 nm. The peak concentration observed during the post-background phase for particles in this size range was 470 particles/cm^3^.

In order to control for the increases observed during the lid opening and post-concentration periods, an administrative control could be implemented whereby the local exhaust ventilation system is left on for a few minutes after the laser cutting is completed. Currently, operators turn off the LEV as soon as the cut is complete and open the lid right away as their common practices. Giving the LEV the opportunity to clear the NPs would serve greatly to reduce the laser-generated contaminants that are released.

### Total Concentration and Distribution in 0.3–10 μm Particles

The opposite trend is seen for particulate matter ranging from 0.3 to 10 μm shown in Figure 1B. In contrast to the gradual increase seen for smaller particles, the concentrations within the size range would normalize almost immediately after peaking. As mentioned previously, the OPS was more susceptible to interferences from the second laser cutter that was operated at certain times during the experimental period that is seen in Figure 1B with clear peaks occurring during the background and laser cutting periods. An ANOVA test identified that there was a significant difference in the means of the background, laser cutting, lid opening, and post-background concentrations in the experimental Methods 2 and 3, but no significant difference between means in Method 1 (Method 1: *p*-value = 0.47; Method 2: *p*-value = 2.42 × 10^–8^; Method 3: *p*-value = 2.95 × 10^–3^).

The distribution of particle sizes monitored by the OPS (0.3–10 μm) remained similar among all experimental methods (Figure S3). The graphs were skewed to the right, with the smallest particles (0.35 μm) having the highest distribution. The peak concentration reached in this size range was 69, 101, and 123 particles/cm^3^ during Methods 1, 2, and 3, respectively, during the post-background period. The same statistical analysis (Pearson’s correlation) was used to compare the particle size distributions during each of the portions of the laser cutting as was used for the SMPS data (Tables S6–S8). The particle size distributions had a high degree of correlation during each phase of the laser cutting activity. This once again shows that the operation or opening of the laser cutter did not result in a change in particle size distributions.

### Total Concentration and Distribution in 0.3–10 μm Particles from a Distance

A set of data was collected using the OPS from a desk ∼7 feet (2.1 m) from the laser cutter. The sample was taken concurrently with another OPS set at a distance 2.5 inches (6.4 cm) from the lid of the laser cutter (Figure S4). The most noticeable observation was that neither the OPS located 2.5 (6.4 cm) inches from the laser cutter nor the one located 7 feet away (2.1 m) measured an increase in total particle concentrations when the lid was opened after the first (primary) laser cutter had completed a 10 min cut. However, a peak in particulate data was observed at both locations during the post-background period, which was a result of the second laser cutter being used. The second laser cutter was also utilized to cut a piece of acrylic, but the process only lasted for about 2 min, followed by a period during which the operator left the lid open for ∼90 s. The distance of both OPSs from this second laser cutter was ∼7 feet (2.1 m) and yet the increase in concentrations was significant, reaching concentrations of 130 particles/cm^3^. Because of this clear spike in concentrations from a nearby laser cutter, future research will focus on setting up multiple instruments at several locations and observe the way that time of cut may influence these concentrations with multiple cutters close to each other.

There were certain limitations that existed with the data that was collected among all experimental trials. The most important to note is that the environment in which data was collected resembled that of a field study that represents the practical and real-life exposure to the operation of laser cutting. Since the facility could not be used outside of the normal hours of operations, the results could also have been influenced by the number of times the door was opened, number of persons within the facility, and operation of other equipment such as laser cutters. Despite these factors, based on the data that was collected by both real-time instruments, most particles that were released by the laser cutting activities were predominantly in the nanometer-sized range. This emphasizes the need to further characterize the particles as discussed in the next section.

### Characterization of Particle Size and Morphology

**Figure 2:**
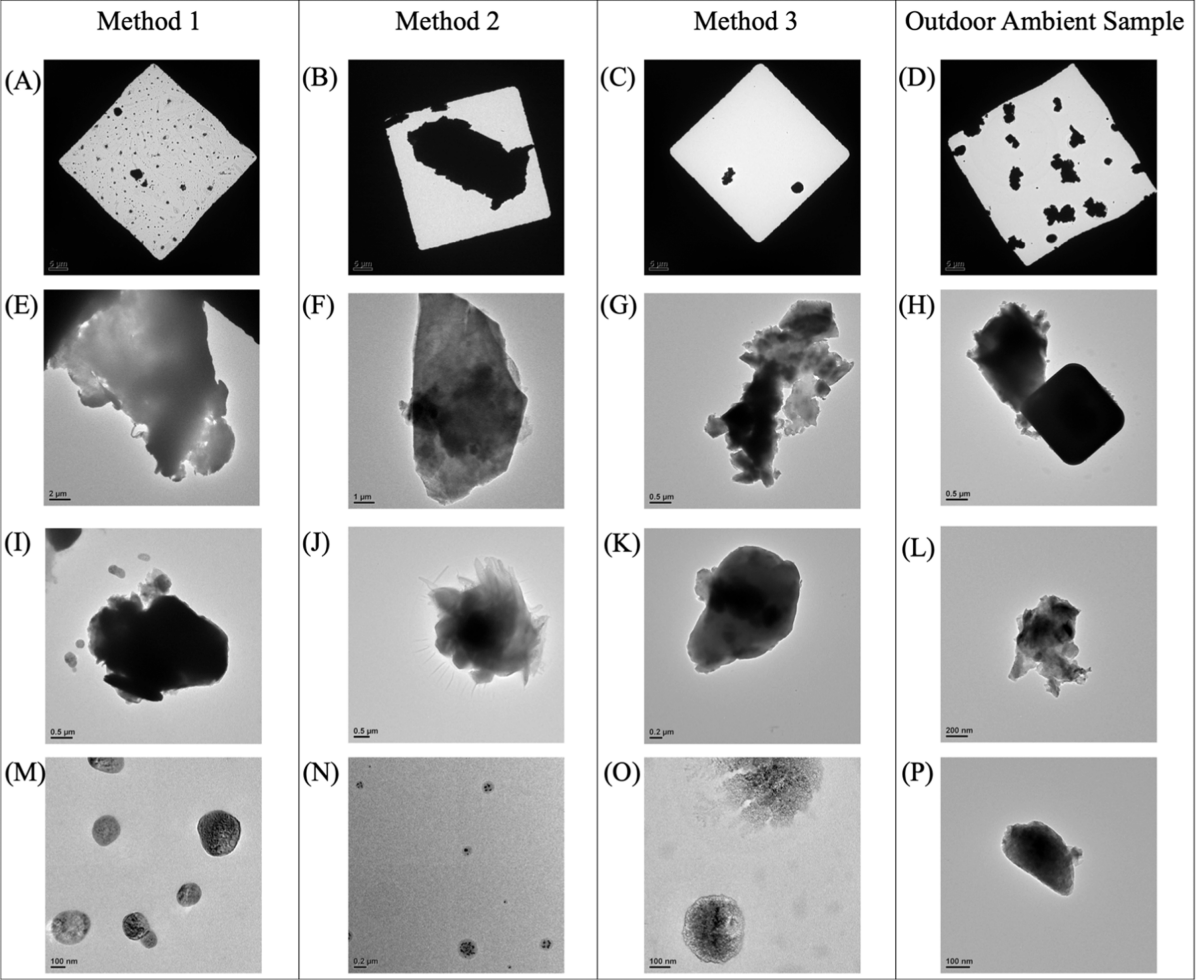
TEM images of the structure and size of particulates were captured using the T12 microscope contained on a carbon-coated, TEM-copper grid that were collected during the experimental periods and an ambient outdoor sampling period. Panels (A–D) represent the range in sizes collected and observed on a single TEM grid during all of the sampling periods. (E–L) Particulates captured among all sampling periods that closely resemble the mineral dust particles by Ott et al. (M–P) Particles captured during all sampling periods with TEM that resemble organic (M, N, O)/inorganic (P)-containing particles. Note: The scale bar is 5 μm in panels (A–D); 2 μm in panel (E); 1 μm in panel (F); 0.5 μm in panels (G–J); 0.2 μm in panels (K, N); 200 nm in panel (L), and 100 nm in panels (M, O, and P). Images (D, H, L, and P) correspond to the collected outdoor ambient air samples. The rest of the images correspond to those collected from the experimental methods.

The airborne particles sampled near the laser cutting processes varied in size and shape, as seen in Figure 2. The images included were representative of particulate matter seen across all of the laser cutting experiments that were conducted. TEM images were presented according to the experimental method during which they were captured. Figure 2A–C was included to illustrate the observed particle concentration and sizes throughout all of the experimental methods. Each experimental method resulted in a high concentration of smaller-sized (nano and submicron) particulates (Figure 2A), while larger-sized (micrometer) particles (Figure 2B) were not as frequent, but still observed. This corresponds with the findings of the distribution graphs discussed earlier, as smaller (nano and submicron) particles were highly distributed.

According to Ott et al., in brightfield imaging, darker images indicate that electrons are not able to pass through the sample, thus indicating that the particle has a higher degree of thickness in comparison to the lighter images.^29^ An example of the varying degrees of thickness captured in the samples could be observed between Figure 2E,I, which were two particles that appear to have similar shapes, but Figure 2I would be considered to be much thicker based on the darkness of the particle.

The particles observed in Figure 2E–G,I–K were representative of the most common type of particulate that was observed in the samples collected in each of the experiments. The particles varied in apparent thickness and diameter with some reaching upward to 10 μm in diameter. They closely resemble particles that are seen and described by Ott et al. as being mineral dust particles, which are made up of a combination of different mineral species.^29,30^ That indicated, particulates captured among all sampling periods that closely resemble mineral dust particles as reported by Ott et al. According to Ott et al., these types of aerosols are the second largest emissions by mass into the Earth’s atmosphere.^29,30^ Therefore, it is likely that some of these particles were collected from the ambient environment.

Another type of particle that appeared within the sample was seen in Figure 2M–O. The circular particles varied in size, with some having a diameter as small as 100 nm. Based on reference images, these appear to have the same characteristics as those described as organic/inorganic-containing particles by Ott et al.^29,30^ However, it is important to note that the true particle size and diameter may have been altered when being observed under the TEM. The surface texture, which appeared to be bubbly (Figure 2M–O), might have been the result of electron beam damage undergone during the magnification of the sample indicating the organic particles.^29^

### Ambient Outdoor Particulates

An additional set of data was collected with an OPS at an outside location 5 feet (1.5 m) from the door of the facility where the laser cutter was located. The instrument measured continuously for a total of 2 h from the hours of 9–11 AM, as this was the time during which the experiments were conducted (Figure S5). During the 2 h period, there were several peaks in total particulate concentration that reached as high as 55 particles/cm^3^ in the size range of 0.3–10 μm. Peaks in the concentration could be attributed to several factors, including the presence of vehicles that pass by frequently to access a nearby warehouse. The particle size distribution was skewed to the right, with particles with 0.35 μm having the highest concentrations. The highest peak concentration in that size range reached 50 particles/cm^3^.

The particles that were observed on the outdoor sample (Figure 2D,H,L,P) had many similarities to those that were captured during the laser cutting activities. The diameter size of the captured particles ranged anywhere from 100 to 10 μm. The samples seen in Figure 2H,L,P also closely resembled the mineral dust particles mentioned in Ott et al.^29,30^ However, there were larger agglomerates that were visible, such as the one seen in Figure 2H, where the attached particle has a square shape.

### STEM-EDX Analysis

**Figure 3:**
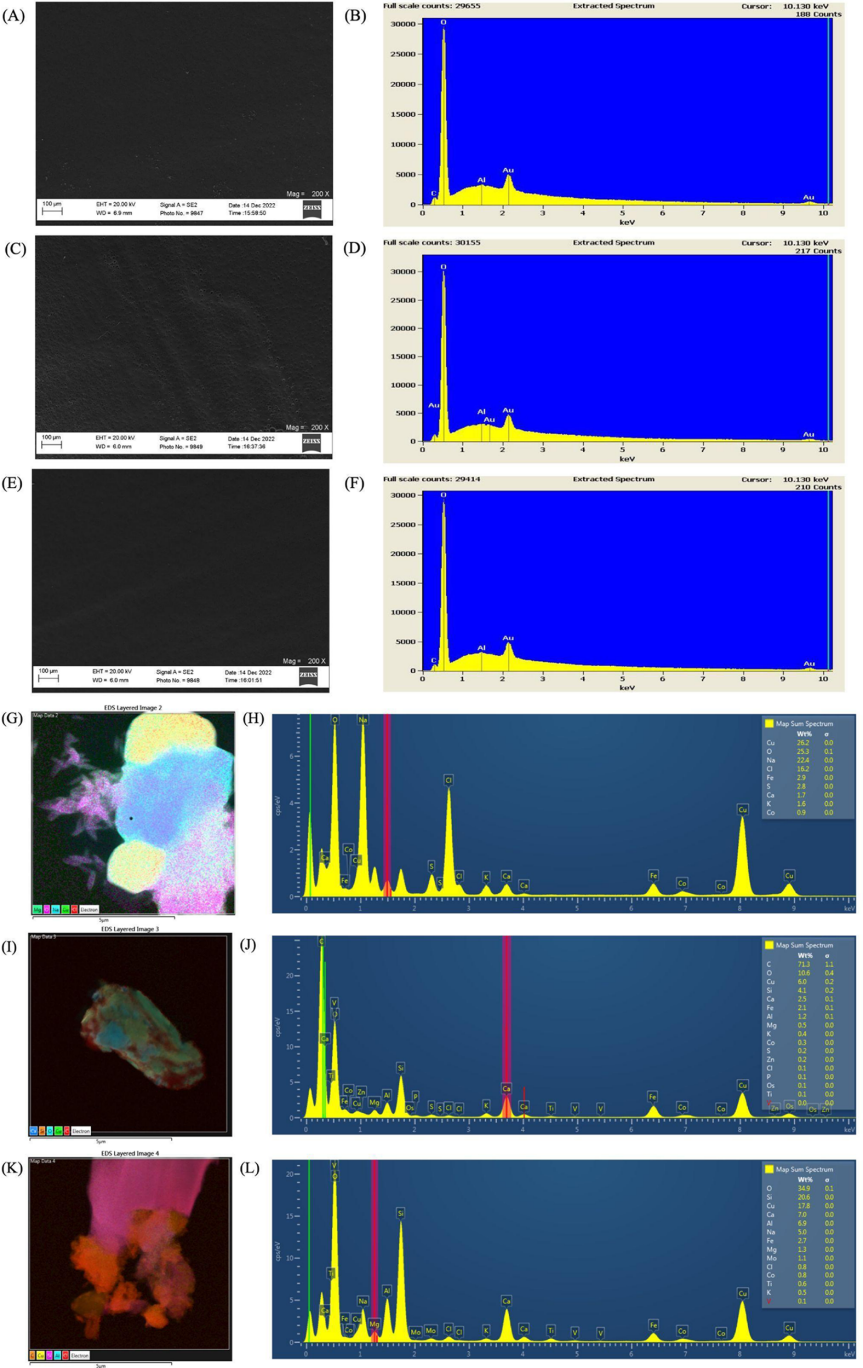
(A–F) Images and elemental composition analysis of fragments of acrylic pieces analyzed using STEM. The fragments detected the presence of two main elements, aluminum and oxygen, which were used as tracer elements in the subsequent aerosol sample analysis. (G–L) Images and elemental composition of several aerosol particles collected in the ambient air after laser cutting had been completed. In panels (I–K), we observe the presence of aluminum and oxygen, as a result of the release of PMMA particles into the air from laser cutting. These particles have agglomerated, resulting in the detection of various other elements in different regions of the particles observed. Note: The scale bar is 100 μm in panels (A, C, and E) and 5 μm in panels (G, I, and K). The primary elements detected in panels (A, C, and E) are C, Al, O, and Au. The primary elements detected in panel (G) are Cu, O, Na, Cl, Fe, S, Ca, K, and Co; the primary elements detected in panel (I) are C, O, Cu, Si, Ca, Fe, Al, Mg, K, Co, S Zn, Cl, P, Os, and Ti; and the primary elements detected in panel (K) are O, Si, Cu, Ca, Al, Na, Fe, Mg, Mo, Cl, Co, Ti, and K.

To further verify the presence of airborne PMMA microplastic particles emitted from laser cutting acrylic, elements contained in the acrylic and airborne particulates were analyzed and compared. Fragments of the acrylic plastic sheet used in laser cutting were scraped at three different spots to obtain three tiny thin pieces, which were analyzed under SEM (Figure 3A,C,E). The elemental compositions of the three fragments also were characterized using energy-dispersive X-ray spectroscopy (EDX). EDX results on these acrylic samples (Figure 3B,D,F) showed the presence of two main elements: oxygen and aluminum.^31−33^ Based on the U.S. patent of acrylic materials, aluminum in the form of alumina trihydrate is an important additive substance in the acrylic materials and likely can be contained in most types of acrylic material because the alumina trihydrate additive provides a higher resistance to heat, stress cracking, and a higher level of translucency.^31−33^ Because aluminum is a distinguished additive contained in acrylic, this was used as the tracer to determine the presence of PMMA microplastics released from acrylic cutting and being collected in our airborne samples.

Airborne particles collected on the TEM grids using the TDS were also subjected to an EDX analysis to identify and verify the sizes and compositions of emitted airborne particles from laser cutting. We hypothesized that we would detect the presence of acrylic nano- and microplastics since the air sample had been collected during and after laser cutting acrylic sheets. As a result of the EDX analysis, particulates ranging from 1 to 10 μm on the TEM grid (Figure 3G,I,K) were found to have varying elemental compositions (shown in Figure 3H,J,L). Most of the larger captured particulates were saturated with metal elements (Mg, Cu, Ca) and might have been captured from the ambient air from other aerosol-generating processes in the area. However, from the spectrum associated with each of the samples (Figure 3H,J,L), the sample was also composed of primarily oxygen and aluminum (gold is from the layer of a sputtercoat required for analysis), which clearly indicated the presence of acrylic/PMMA microplastic particles released from acrylic. As discussed, since aluminum is a common additive within acrylic materials, we can conclude that these particles contained PMMA particles, known as microplastics and nanoplastics, that were being released into the air as a result of laser cutting.^31−33^ The other elements found on the particles could be contaminants in the background air such as diesel particles, which would be found in the workplace due to the area in which this workplace was located. Based on Figure 3G,K, it indicated that PMMA particulate matter released from laser cutting, and some have agglomerated with ambient particles, such as diesel, found in the air. According to OSHA, diesel particulate matter is made up of primarily carbon ash, metallic abrasion particles, sulfates, and silicates.^34,35^Figure 3J,L shows the heavy presence of these elements, in addition to the aluminum and oxygen that were the primary elements contained for PMMA.

It is important to note that small nano and submicron particulates on the filmed grid substrate were clearly seen on the microscopy analysis and detected by real-time instruments. Our study was conducted based on short time cutting activities; a prolonged operation and repetitive cutting activities during an 8 h work shift would emit a much higher level of MMA and plastic particles.

### Gas Sampling and Chromatography

Gas samples were collected on two separate occasions using a manual pump and a 1 L Teflon gas bag (Jensen Inert Products, Coral Springs, FL). The first sample was collected while no laser cutting activities were in progress as a reference sample. The second gas sample was collected during the lid opening portion of the laser activity. Using the analytical method described in the experimental process, there were no identifiable chemical contaminants found in the background sample. During the lid opening portion, the chemical MMA was the only chemical that was identified (CAS 80-26-6). Methyl methacrylate (MMA) that has a plastic odor with a retention time of 5.7 min was identified as the major component in the sample by comparison of the acquired mass spectrum from a NIST library of mass spectrum.^36,37^ The probability was 81%. Confirmation of the MMA was complete in the following manner. A standard MMA was placed in a 1 L Teflon Bag and injected as above. The retention time matches the sample identified by mass spectroscopy at 5.7 min.

MMA is an organic compound that is formed when PMMA undergoes thermal degradation.^38^ Exposure to high concentrations of MMA can lead to irritation of the skin, eyes, and mucous membranes in humans.^38^ Additionally, chronic inhalation has been documented to result in respiratory and nasal symptoms, reduced lung function, and even cardiovascular disorders in humans.^38^ Since the chemical was identified during gas sampling, it signified that gas emissions were being released during the lid opening portion of laser activities, despite the use of a fume extractor during the process. MMA is described as having an acrid, repulsive odor, which was noticed during the laser cutting activity.^28^

**Figure 4:**
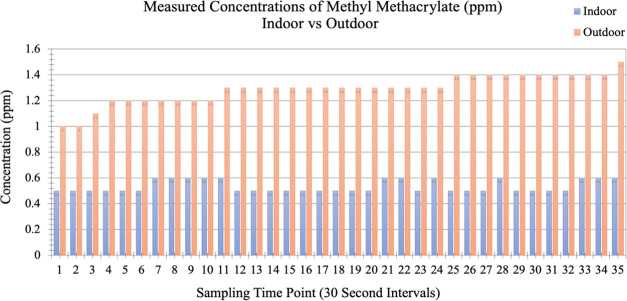
Concentrations of methyl methacrylate (MMA) in ppm present in indoor environments during laser cutting activities compared to the outside ambient air.

Through a follow-up analysis of the ambient air using the portable IR analyzer measuring the entire period of laser cutter operation including lid opening as shown as indoor data in Figure 4, it was discovered that despite the strong odor released from laser cutting PMMA, the concentrations of MMA were at or below the detectable range (0.4 ppm). The strong odor that was observed was attributed to the relatively low odor threshold of MMA, which is 0.08 ppm.^38^ To compare with the MMA concentrations outside of the facility, the IR analyzer was used to sample the outside ambient air as well (5 feet [1.5 m] away from the entrance) as shown as outdoor data in Figure 4. The comparisons between the measured concentrations of MMA in the indoor and outdoor environment are shown in Figure 4.

Despite MMA being identified using the preliminary GC-MS analysis, the concentrations that were measured, if operated for a work shift under the same conditions, were well below the 100 ppm permissible exposure limit (PEL) set by the Occupational Safety and Health Administration (OSHA). The measured indoor concentrations (mean—0.5 ppm) and the outside concentrations (mean—1.3 ppm) were both considered to be well below the OSHA PEL. Based on the outside air concentrations, it was possible that the indoor levels of MMA could have also been brought in from the outside. Indoor MMA concentrations could have been a result of the frequent door opening that occurred, or from the ventilation system itself. The air duct for the ventilation system, which uses outside air, faces an industrialized area and is located at the floor level. Contaminants generated from the outside could be sucked into the facility via the ventilation system. Regardless of where the MMA was produced, it can be concluded that the gas emissions released by the laser cutter were not at a concentration high enough to cause concern under the studied operating conditions.

## Conclusions

As a result of this study, it was determined that the fume extractor was efficient at capturing the gas emissions produced from the laser cutting PMMA material, but not the particulates such as microplastics. Previous studies had reported concern about MMA emission, yet our results revealed the MMA emission was close to the detection level, showing that the fume extractor was efficient at capturing the chemical gases.^5,24^ The opening of the lid resulted in high peak concentrations of fine and ultrafine particulate matter, which could deposit deep into the lungs and might contribute to potential health problems. Previous studies had shown that particulate emissions for laser cutting PMMA were not as high as other types of plastics, yet there was still a significant increase found even after the usage of the fume extractor.^5,24^ The amount of particulate matter released would vary on factors such as the cut time and design, and more research would be needed to determine which cut style may produce the highest concentration of particulates. One potential administrative control that can be applied is to keep the fume extractor on longer after the cut has been completed in order to give it time to fully filter the contaminants that are being produced by the laser cutting activity. Currently, operators of the laser cutters typically turn off the LEV as soon as the cut is complete and open up the lid right after. The study results provided evidence supporting our recommendation to the workplace to change the process to allow the LEV to continue running for a longer period of time after the cut is complete, allowing all of the airborne emissions/particles to be cleared from the system. It was noticed that during each of the experiments, during the laser cutting portion, the concentrations of particulate matter would stay at a constant level, likely contributed by the use of a fume extractor. It was not until the fume extractor was turned off that the concentration of particulate matter, especially those in the nanometer-sized range, began to gradually increase. The results indicated that despite the use of the fume extractor, significant concentrations of particulates with a majority in the sizes of the peaks at 27.4–36.4 nm above 2821 and 3057 particles/cm^3^ (for Methods 1 and 3, respectively) in concentration were being released after the lid was opened to retrieve the finished product. The emitted airborne particles were confirmed to contain acrylic and appeared in the form of microplastic particles. Special attention should be paid to particle concentrations during the time period after the laser cutting has been completed, especially if the laser cutter is used frequently. These nanometer-sized particles appear to have a delayed release from the laser cutter and can continuously increase in concentration if proper ventilation systems are not in place.

## Supporting Information Available

Experimental setup photo, particle size fractioned concentrations and total concentrations measured by the OPS, experimental time period of laser cutting, statistical analysis results presenting correlation analysis, ANOVA, and *t*-test results of SMPS/OPS data (PDF)

## Abbreviations

LGACs: laser-generated air contaminants
TDS: Tsai diffusion sampler
GC-MS: gas chromatograph-mass spectrometry
PAHs: polycyclic aromatic hydrocarbons
nm: nanometers
μm: micrometers
NPs: nanoparticles
PMMA: poly(methyl methacrylate)
MMA: methyl methacrylate
CO_2_: carbon dioxide
HEPA Filter: high-efficiency particulate air filter
SMPS: scanning mobility particle sizer
OPS: optical particle sizer
AIM: aerosol instrument manager
PC: polycarbonate
TEM: transmission electron microscopy
SPME: solid phase microextraction
PLA: poly(lactic acid)
ABS: acrylonitrile butadiene styrene
ANOVA: analysis of variance
IR: infrared
OSHA: Occupational Safety and Health Administration
PEL: permissible exposure limit
CNSI: California NanoSystems Institute
