# Supplementary material for: Characterization of Emissions from Carbon Dioxide Laser Cutting Acrylic Plastics
Source: J Chem Health Saf. 2023 Jun 22;30(4):182–92. doi: 10.1021/acs.chas.3c00013 (PMC10369487; doi:10.1021/acs.chas.3c00013)
Supplement: Supplementary file 1 — hs3c00013_si_001.pdf [file hs3c00013_si_001.pdf]

Supporting Information

Characterization of Emissions from Carbon Dioxide Laser Cutting Acrylic Plastics

Alejandro Munoz[1], Jacob Schmidt[2], I.H. (Mel) Suffet[1], Candace Su-Jung Tsai[1]*

[1] Department of Environmental Health Sciences, Fielding School of Public Health, University of California, Los Angeles, Los Angeles, California, 90095-1735, United States

[2] Samueli School of Engineering, University of California, Los Angeles, Los Angeles, California, 90095-1735, United States

* Corresponding author: Dr. Candace S.J. Tsai,

Email: candacetsai@ucla.edu

Affiliation: Department of Environmental Health Sciences, Fielding School of Public Health, University of California, Los Angeles, Los Angeles, California, 90095-1735, United States

Address: 650 Charles E. Young Drive S., MC 177220, Los Angeles, California 90095-1735, United States

Phone: (310) 206-9258

TABLE OF CONTENT

Figure S1. Experimental setup for the monitoring of particle emissions: (A) front-facing view with the two instruments NanoScan SMPS and OPS placed in front of the laser cutter. (B) Side view showing the distance of the Tygon tubing from the lid of the laser cutter lid.

Figure S2. The total concentration of particulate matter during the four different periods of the experimentation was (1) background, (2) laser cutting, (3) lid opening, and (4) post background for particulates with diameters ranging from 10-420 nm as measured by the NanoScan SMPS.

Figure S3. Particle size fractioned concentrations (particles/cm^3) measured by the OPS during each phase of the experimental method (A) Size distribution for Method 1 (B) Size distribution for Method 2 (C) Size distribution for Method 3.

Figure S4. The total concentration of particulate matter during the four different periods of the experimentation was (1) background, (2) laser cutting, (3) lid opening, and (4) post background for particulates with diameters ranging from 0.3-10 µm as measured by 2 OPSs at a distance of 2.5 inches and 7 feet concurrently.

Figure S5. The total concentration and particle size distribution of particulates were measured outside of the facility using the Optical Particle Sizer (TSI OPS Model 3330). (A) Total particle concentration trends throughout the two hours monitoring period. (B) Particle size distributions of the particulate matter ranging from 0.3-10 µm in diameter.

Table S1. Experimental methods with time periods that were used to monitor the fugitive emissions from laser cutting.

Table S2. Date and time each of the experimental trials were performed.

Table S3. Correlation analysis between the background, laser cutting, lid opening, and post background of the SMPS data for experimental method 1.

Table S4. Correlation analysis between the background, laser cutting, lid opening, and post background of the SMPS data for experimental method 2.

Table S5. Correlation analysis between the background, laser cutting, lid opening, and post background of the SMPS data for experimental method 3.

Table S6. Correlation analysis between the background, laser cutting, lid opening, and post background of the OPS data for experimental method 1.

Table S7. Correlation analysis between the background, laser cutting, lid opening, and post background of the OPS data for experimental method 2.

Table S8. Correlation analysis between the background, laser cutting, lid opening, and post background of the OPS data for experimental method 3.

Table S9. Analysis of Variance (ANOVA) between total concentration means within the experimental methods. Note: Group mean refers to the average total concentration of

each of the phases of the experimental method (i.e., background, laser cutting, lid opening, and post-background)

Table S10. Two sample t-test between background and post-background concentrations for each experimental method (assuming equal variance).

SUPPORTING INFORMATION

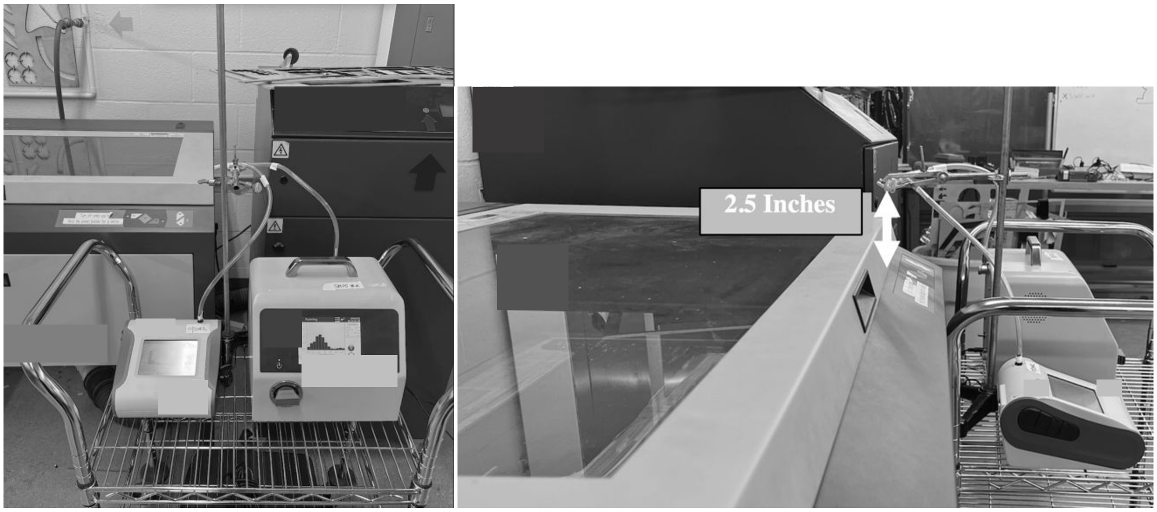

Figure S1. Experimental setup for the monitoring of particle emissions: (A) front-facing view with the two instruments NanoScan SMPS and OPS placed in front of the laser cutter. (B) Side view showing the distance of the Tygon tubing from the lid of the laser cutter lid.

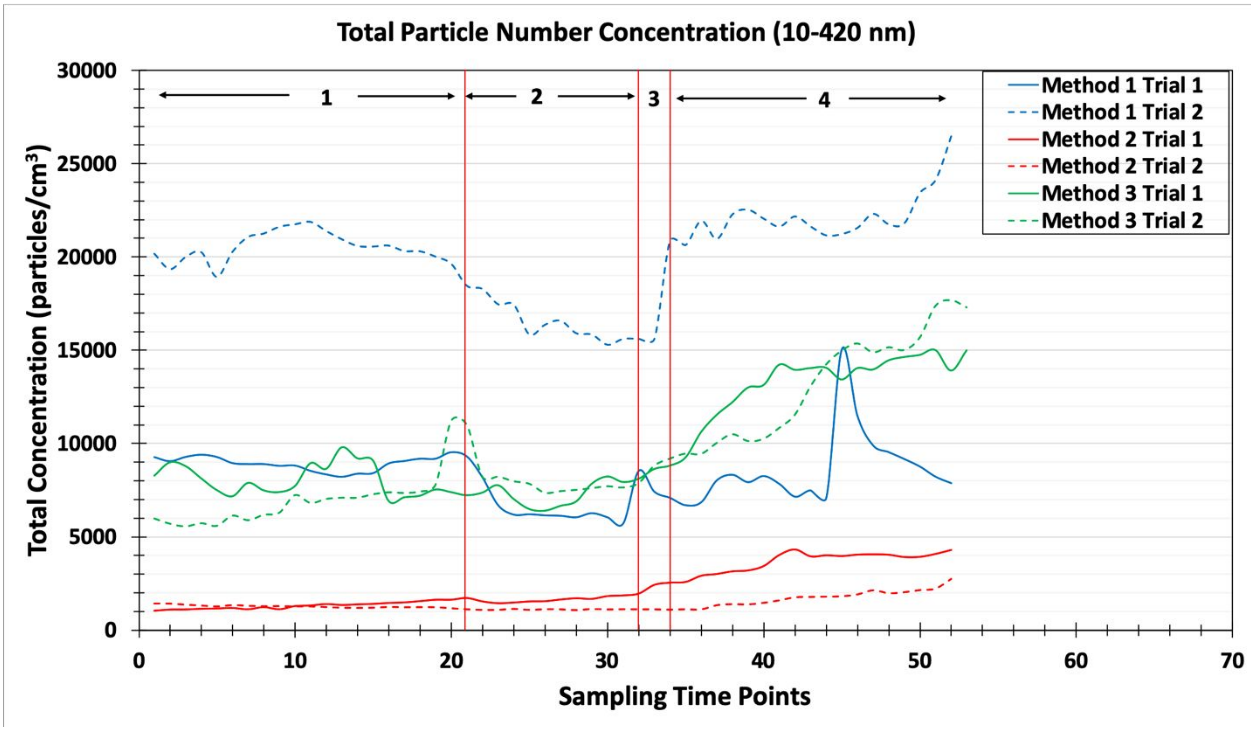

Figure S2. The total concentration of particulate matter during the four different periods of the experimentation was (1) background, (2) laser cutting, (3) lid opening, and (4) post background for particulates with diameters ranging from 10-420 nm as measured by the NanoScan SMPS.

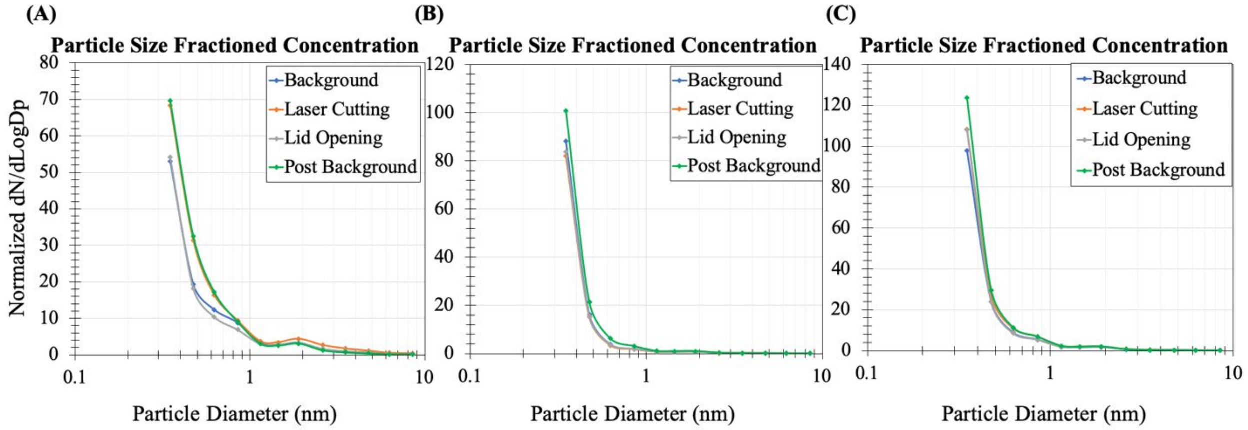

Figure S3. Particle size fractioned concentrations (particles/cm^3) measured by the OPS during each phase of the experimental method (A) Size distribution for Method 1 (B) Size distribution for Method 2 (C) Size distribution for Method 3.

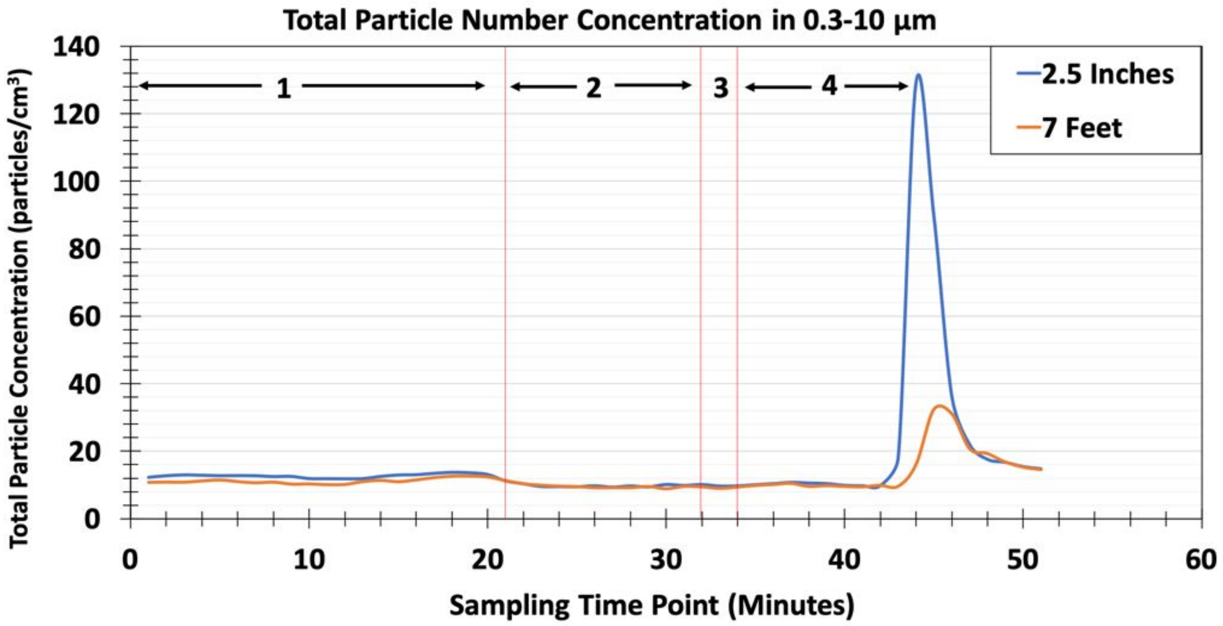

Figure S4. The total concentration of particulate matter during the four different periods of the experimentation was (1) background, (2) laser cutting, (3) lid opening, and (4) post background for particulates with diameters ranging from 0.3-10 µm as measured by 2 OPSs at a distance of 2.5 inches and 7 feet concurrently.

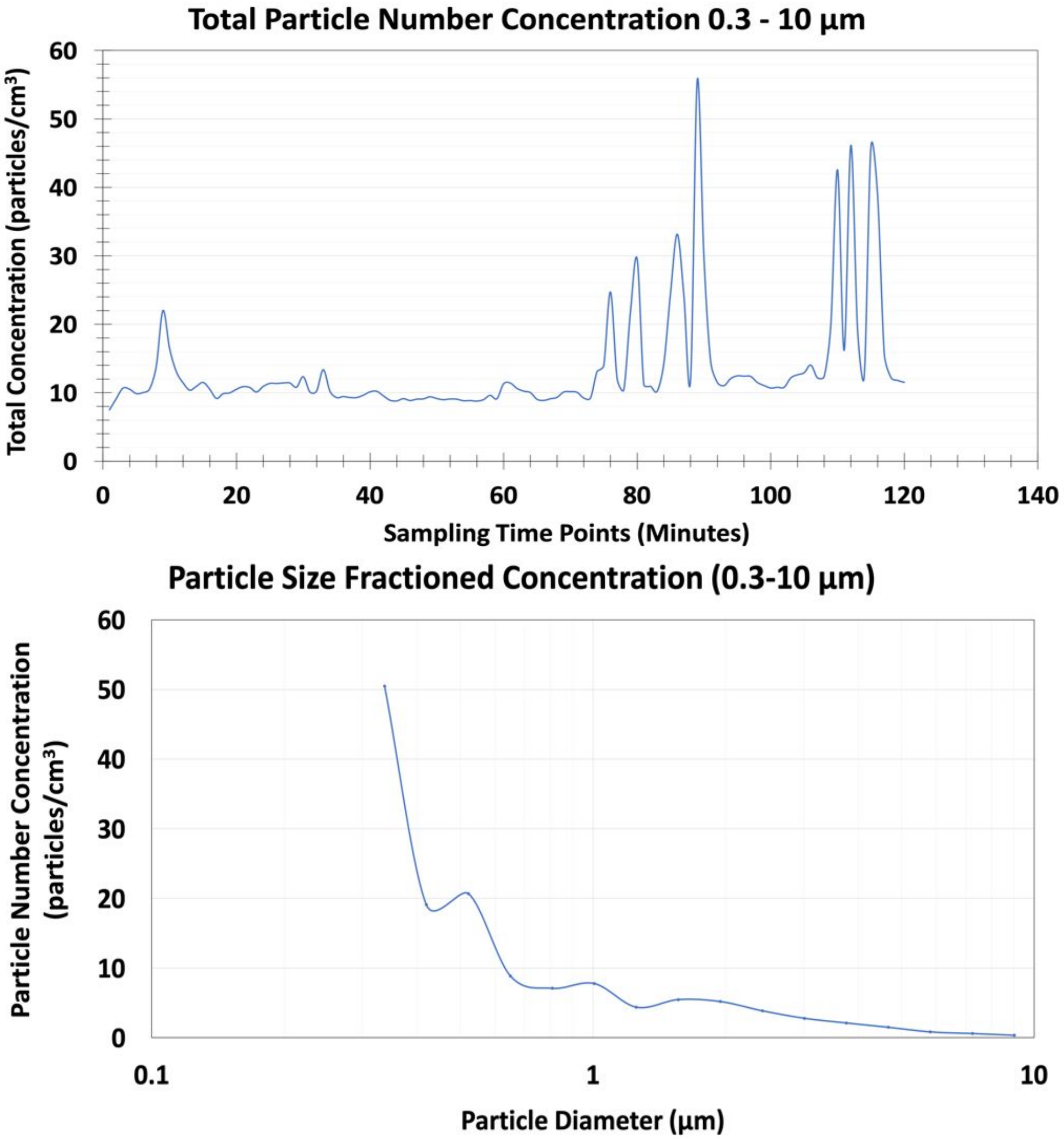

Figure S5. The total concentration and particle size distribution of particulates were measured outside of the facility using the Optical Particle Sizer (TSI OPS Model 3330). (A) Total particle concentration trends throughout the two hours monitoring period. (B) Particle size distributions of the particulate matter ranging from 0.3-10 µm in diameter.

Table S1. Experimental methods with time periods that were used to monitor the fugitive emissions from laser cutting.

Method	Background	Cutting Time	Time Waited Before Turning off Fume Extractor and Opening Lid	Time Lid Was Left Open	Post Background
1	20 Minutes	10 Minutes	0 Seconds	30 Seconds	20 Minutes
2	20 Minutes	10 Minutes	30 Seconds	30 Seconds	20 Minutes
3	20 Minutes	10 Minutes	1 Minute	30 Seconds	20 Minutes

Table S2. Date and time each of the experimental trials were performed.

Experiment	Date	Start Time	Average Background Concentration
Method 1<br>*Trial 1*	May 10th, 2022<br>*(Tuesday)*	9:17 AM	8,925.6 particles/cm^3
Method 1<br>*Trial 2*	May 9th, 2022<br>*(Monday)*	10:13 AM	20,544.7 particles/cm^3
Method 2<br>*Trial 1*	May 3rd, 2022<br>*(Tuesday)*	9:17 AM	1,308.0 particles/cm^3
Method 2<br>*Trial 2*	May 5th, 2022<br>*(Thursday)*	9:16 AM	1,273.6 particles/cm^3
Method 3<br>*Trial 1*	May 5th, 2022<br>*(Thursday)*	10:17 AM	8,051.7 particles/cm^3
Method 3<br>*Trial 2*	May 9th, 2022<br>*(Monday)*	9:18 AM	6,841.2 particles/cm^3

Table S3. Correlation analysis between the background, laser cutting, lid opening, and post background of the SMPS data for experimental method 1.

**Method 1- SMPS**		**Background**	**Laser Cutting**	**Lid Opening**	**Post Background**
**Background**	*Pearson*	1.000	0.998	0.994	0.999
	Sig.	2.2 E -16	4.9 E - 14	6.8 E -12	2.2 E -16
	N	13	13	13	13
**Laser Cutting**	*Pearson*	0.998	1.00	0.995	0.998
	Sig.	4.9 E - 14	2.2 E -16	1.4 E -12	9.6 E -15
	N	13	13	13	13
**Lid Opening**	*Pearson*	0.994	0.995	1.00	0.993
	Sig.	6.8 E -12	1.4 E -12	2.2 E -16	2.0 E -11
	N	13	13	13	13
**Post Background**	*Pearson*	0.999	0.998	0.993	1.00
	Sig.	2.2 E -16	9.6 E -15	2.0 E -11	2.2 E -16
	N	13	13	13	13

Sig. presenting the p-value of analysis in Table S2-S7.

Table S4. Correlation analysis between the background, laser cutting, lid opening, and post background of the SMPS data for experimental method 2.

Method 2 - SMPS		Background	Laser Cutting	Lid Opening	Post Background
**Background**	*Pearson*	1.00	0.920	0.801	0.500
	Sig.	2.2 E -16	8.5 E -6	9.9 E -4	0.08
	N	13	13	13	13
**Laser Cutting**	*Pearson*	0.920	1.00	0.968	0.773
	Sig.	8.5 E -6	2.2 E -16	6.4 E -8	1.9 E -3
	N	13	13	13	13
**Lid Opening**	*Pearson*	0.801	0.968	1.00	0.892
	Sig.	9.9 E -4	6.4 E -8	2.2 E -16	4.2 E -5
	N	13	13	13	13
**Post Background**	*Pearson*	0.500	0.773	0.892	1.00
	Sig.	0.08	1.9 E -3	4.2 E -5	2.2 E -16
	N	13	13	13	13

Table S5. Correlation analysis between the background, laser cutting, lid opening, and post background of the SMPS data for experimental method 3.

**Method 3 - SMPS**		**Background**	**Laser Cutting**	**Lid Opening**	**Post Background**
**Background**	*Pearson*	1.00	0.994	0.995	0.993
	Sig.	2.2 E -16	6.7 E -12	2.7 E -12	1.5 E -11
	N	13	13	13	13
**Laser Cutting**	*Pearson*	0.994	1.00	0.997	0.991
	Sig.	6.7 E -12	2.2 E -16	1.6 E -13	6.8 E - 11
	N	13	13	13	13
**Lid Opening**	*Pearson*	0.995	0.997	1.00	0.997
	Sig.	2.7 E -12	1.6 E -13	2.2 E -16	1.0 E -13
	N	13	13	13	13
**Post Background**	*Pearson*	0.993	0.991	0.997	1.00
	Sig.	1.5 E -11	6.8 E - 11	1.0 E -13	2.2 E -16
	N	13	13	13	13

Table S6. Correlation analysis between the background, laser cutting, lid opening, and post background of the OPS data for experimental method 1.

**Method 1 - OPS**		**Background**	**Laser Cutting**	**Lid Opening**	**Post Background**
**Background**	*Pearson*	1.00	0.995	0.998	0.994
	Sig.	2.2 E -16	2.1 E -11	9.8 E -14	6.8 E -11
	N	12	12	12	12
**Laser Cutting**	*Pearson*	0.995	1.00	0.993	0.999
	Sig.	2.1 E -11	2.2 E -16	1.8 E -10	2.2 E -16
	N	12	12	12	12
**Lid Opening**	*Pearson*	0.998	0.993	1.00	0.990
	Sig.	9.8 E -14	1.8 E -10	2.2 E -16	6.9 E -10
	N	12	12	12	12
**Post Background**	*Pearson*	0.994	0.999	0.990	1.00
	Sig.	6.8 E -11	2.2 E -16	6.9 E -10	2.2 E -16
	N	12	12	12	12

Table S7. Correlation analysis between the background, laser cutting, lid opening, and post background of the OPS data for experimental method 2.

**Method 2 - OPS**		**Background**	**Laser Cutting**	**Lid Opening**	**Post Background**
**Background**	*Pearson*	1.00	0.999	0.999	0.999
	Sig.	2.2 E -16	2.2 E -16	2.2 E -16	3.8 E -16
	N	12	12	12	12
**Laser Cutting**	*Pearson*	0.999	1.00	0.999	0.999
	Sig.	2.2 E -16	2.2 E -16	2.2 E -16	3.3 E -16
	N	12	12	12	12
**Lid Opening**	*Pearson*	0.999	0.999	1.00	0.999
	Sig.	2.2 E -16	2.2 E -16	2.2 E -16	3.2 E -16
	N	12	12	12	12
**Post Background**	*Pearson*	0.999	0.999	0.999	1.00
	Sig.	3.8 E -16	3.3 E -16	3.2 E -16	2.2 E -16
	N	12	12	12	12

Table S8. Correlation analysis between the background, laser cutting, lid opening, and post background of the OPS data for experimental method 3.

**Method 3 - OPS**		**Background**	**Laser Cutting**	**Lid Opening**	**Post Background**
**Background**	*Pearson*	1.00	0.999	0.999	0.999
	Sig.	2.2 E -16	2.2 E -16	2.2 E -16	2.2 E -16
	N	12	12	12	12
**Laser Cutting**	*Pearson*	0.999	1.00	0.999	0.999
	Sig.	2.2 E -16	2.2 E -16	8.2 E -16	2.2 E -16
	N	12	12	12	12
**Lid Opening**	*Pearson*	0.999	0.999	1.00	0.999
	Sig.	2.2 E -16	8.2 E -16	2.2 E -16	2.2 E -16
	N	12	12	12	12
**Post Background**	*Pearson*	0.999	0.999	0.999	1.00
	Sig.	2.2 E -16	2.2 E -16	2.2 E -16	2.2 E -16
	N	12	12	12	12

Table S9. Analysis of Variance (ANOVA) between total concentration means within the experimental methods. Note: Group mean refers to the average total concentration of each of the phases of the experimental method (i.e., background, laser cutting, lid opening, and post-background)

	Degrees of Freedom	P-value	F-crit
Method 1 SMPS	3	1.748E-11	2.79806064
Method 2 SMPS	3	4.4114E-17	2.79806064
Method 3 SMPS	3	3.9633E-16	2.79394885
Method 1 OPS	3	0.47287749	2.79806064
Method 2 OPS	3	2.4197E-08	2.79806064
Method 3 OPS	3	0.00295071	2.79394885

Table S10. Two sample t-test between background and post-background concentrations for each experimental method (assuming equal variance).

	Degrees of Freedom	P-value (one tail)	P-value (two-tailed)
Method 1 SMPS	38	0.09845998	0.19692
Method 2 SMPS	38	2.25344E-14	4.50688E-14
Method 3 SMPS	38	1.8248E-13	3.6496E-13
Method 1 OPS	38	0.12476186	0.24952373
Method 2 OPS	38	2.8625E-06	5.7249E-06
Method 3 OPS	38	0.00047066	0.00094132
